# Response to the letter to the editor: “Design and analytical issues: a response to ‘Long-term effects of tongue piercing—a case control study’”

**DOI:** 10.1007/s00784-012-0827-9

**Published:** 2012-08-19

**Authors:** Dirk Ziebolz, Aick Hildebrand, Peter Proff, Sven Rinke, Else Hornecker, Rainer F. Mausberg, Hans-Joachim Helms

**Affiliations:** 1 Department of Preventive Dentistry, Periodontology and Cariology, University Medical Centre Goettingen, Robert-Koch-Str. 40, 37075 Goettingen, Germany; 2 German Army Dental Office, Rotenburg a.d. Fulda, Germany; 3 Department of Orthodontics, University of Regensburg, Regensburg, Germany; 4 Private Practice, Hanau, Germany; 5 Department of Medical Statistics, University Medical Centre Goettingen, Goettingen, Germany

We are pleased that our publication found your interest and we considered your letter. Accordingly, here is our response to the letter to the editor, “Design and analytical issues: a response to ‘Long-term effects of tongue piercing—a case control study.’”

## Point I

It is right that in this case a cohort study was performed and not a case–control study because the endpoints were dental problems and not if the participants have got a piercing [1]. Therefore, we are deeply concerned and thankful. We apologize for this mistake.

## Point II

With respect to the second point in the letter, we point out that the participants were only matched by age because a subgroup of female subjects with piercing was too small to be considered in the analysis (as discussed in the paper). Therefore, only male participants were included in the study (piercing and control), and female participants were dismissed by the study design [1].

In order to reduce an age-dependent effect regarding the dental outcome, a 1:1 control group without piercing yet with corresponding age was performed out of 1,789 male participants by random selection. The range of the age of all participants was 9 years (18 to 27) and the range of all participants with piercing (*n* = 46) was 7 years (19 to 26); there could not be a big impact of the age anyway. However, we are aware of the fact that a 1:1 matching was not really necessary and a 1:2 or 1:3 matching would have been possible, too. The best way would have been to select the control participants only randomly without matching.

However, age was the only matching factor, the range of age was only 9 years and we used a pool of almost 1,839 male persons; we think it is reasonable to consider the control as independent from the exposure group in terms of dental outcome.

We are aware of your remarks, but under the described design, our decision to analyze the data with statistical methods for unpaired data like unpaired *t* tests as well as chi-square tests is also acceptable, according to our statistical advisor (HJH). Furthermore, the statistical analysis with the McNemar’s test and paired *t* test as suggested in the letter showed same results.

In general, it is correct that matched groups are dependent per definition. In the future, for this kind of design we will avoid matching.

We appreciate your letter and your concerns and we hope that this reply will clean out some of your concerns.
